# Monkey Viperin Restricts Porcine Reproductive and Respiratory Syndrome Virus Replication

**DOI:** 10.1371/journal.pone.0156513

**Published:** 2016-05-27

**Authors:** Jianyu Fang, Haiyan Wang, Juan Bai, Qiaoya Zhang, Yufeng Li, Fei Liu, Ping Jiang

**Affiliations:** 1 College of Veterinary Medicine, Nanjing Agricultural University, Nanjing, 210095, China; 2 Jiangsu Co-innovation Center for Prevention and Control of Important Animal Infectious Diseases and Zoonosis, Yangzhou, China; University of Tennessee Health Science Center, UNITED STATES

## Abstract

Porcine reproductive and respiratory syndrome virus (PRRSV) is an important pathogen which causes huge economic damage globally in the swine industry. Current vaccination strategies provide only limited protection against PRRSV infection. Viperin is an interferon (IFN) stimulated protein that inhibits some virus infections via IFN-dependent or IFN-independent pathways. However, the role of viperin in PRRSV infection is not well understood. In this study, we cloned the full-length monkey viperin (mViperin) complementary DNA (cDNA) from IFN-α-treated African green monkey Marc-145 cells. It was found that the mViperin is up-regulated following PRRSV infection in Marc-145 cells along with elevated *IRF-1* gene levels. IFN-α induced mViperin expression in a dose- and time-dependent manner and strongly inhibits PRRSV replication in Marc-145 cells. Overexpression of mViperin suppresses PRRSV replication by blocking the early steps of PRRSV entry and genome replication and translation but not inhibiting assembly and release. And mViperin co-localized with PRRSV GP5 and N protein, but only interacted with N protein in distinct cytoplasmic loci. Furthermore, it was found that the 13–16 amino acids of mViperin were essential for inhibiting PRRSV replication, by disrupting the distribution of mViperin protein from the granular distribution to a homogeneous distribution in the cytoplasm. These results could be helpful in the future development of novel antiviral therapies against PRRSV infection.

## Introduction

Porcine reproductive and respiratory syndrome virus (PRRSV) has caused huge economic loss in the global swine industry [1–3]. Current vaccination strategies and antiviral drugs cannot effectively control PRRSV infection [4]. PRRSV belongs to the family *Arteriviridae*, order *Nidovirales* and is divided into European and North American genotypes based on genetic differences. The PRRSV genome is single-stranded positive-sense RNA and consists of ten open reading frames (ORFs) [5–8]. Among them, ORF1a and ORF1b encode proteins with replicase and polymerase activities. And ORFs 2–7 encode GP2a, GP2b, GP3, GP4, GP5, GP5a, matrix protein (M) and nucleocapsid protein (N) [9–14], which are related to the process of viral entry, assembling, and release.

The innate immune response is the first line of defense against infections. Pattern recognition receptors (PRRs) are essential for the detection of pathogen-associated molecular patterns (PAMPs). Toll-like receptors (TLRs) 3, 7–9, retinoic acid inducible gene-I (RIG-I), melanoma differentiation-associated gene-5 (MDA5), STING, POIIII, DDX41, DAI, IFI16, AIM and cGAS recognize foreign nucleic acids to activate type I interferon (IFN) production pathway. And then IFNs are secreted outside the cell to bind the IFN receptors on itself or neighbor cells. The Janus kinase (JAK) and Tyk2 are activated by receptor binding to phosphorylate the signal transducers and activators of transcription 1(STAT1) and 2. Phosphorylated STAT1 and STAT2 along with IRF9 form the ISGF3 complex, and the ISGF3 complex is translocated into the nucleus to bind IFN-stimulated response elements (ISREs), which results in the induction of numerous IFN-stimulated genes (ISGs) that interfere with multiple stages of the virus life cycle and limit viral infection [15, 16]. But the exact molecular mechanisms of specific ISGs to combat different pathogens are not fully understood. Viperin (also known as RSAD2) is an ISG that is found in most tissues and cells at a very low basal level and is induced by multiple viruses via IFN-independent and IFN-dependent pathways [17, 18]. Viperin is localized to the endoplasmic reticulum (ER) membrane and lipid droplets via its N-terminal amphipathic α-helix [19–21]. It has been shown to have anti-viral actions in many viral infections such as hepatitis C virus (HCV) [22, 23], influenza virus [24, 25], human immunodeficiency virus (HIV) [26, 27], equine infectious anemia virus (EIAV) [28], Dengue virus (DENV) [29, 30], sindbis virus (SINV) [31], Japanese encephalitis virus (JEV) [32], West Nile virus (WNV) [30, 33], Bunyamwera virus [34], respiratory syncytial virus (RSV) [35, 36], tick-borne encephalitis virus (TBEV) [37], and Chikungunya virus [38–40]. In this study, it was first found that PRRSV and IFN-α both induced the expression of monkey viperin (mViperin) in Marc-145 cells. Over-expression of mViperin could inhibit PRRSV replication in a dose-dependent manner. Moreover, it blocked PRRSV entry and genome replication and translation but had no effect on assembly and release of PRRSV in Marc-145 cells. Meanwhile, mViperin can co-localize with PRRSV GP5 and N protein, and interact with N protein *in vitro*.

## Materials and Methods

### Viruses and cells

Marc-145 cells, a subclone of African green monkey kidney MA104 cells, and BHK21 cells were purchased from American ATCC and maintained in Dulbecco’s Modified Eagle’s medium (DMEM; GIBCO, Shanghai, China) with 10% fetal bovine serum (FBS; GIBCO) penicillin (100 U/mL) and streptomycin (100 μg/mL) at 37°C in 5% CO_2_. HP-PRRSV BB0907 strain (GenBank accession number: HQ315835) was isolated from a clinically diseased pig in Guangxi Province, China, in 2009 and propagated in the Marc-145 cells, and a 50% tissue culture infection dose (TCID50) was determined by titration in Marc-145 cells. PRRSV strain BB0907 infectious cDNA clone were constructed and preserved in our laboratory.

### Effect of IFN-α on induction of mViperin

To investigate the induction of mViperin by IFN-α, Marc-145 cells were seeded onto 12-well plates for 24 h and treated with IFN-α (Peprotech, Rocky Hill, USA) at various concentrations of 0, 300, 1000 and 3000 U/mL, respectively. Twenty-four hours later, the cells were challenged with PRRSV at 0.1 multiplicity of infection (MOI). After being incubated for 12, 24, 36 and 48 h, the cells were harvested, and both mViperin and PRRSV were detected by real-time PCR and western blotting [41].

### Construction of plasmids expressing full-length and truncated mViperin

Total RNA was extracted from Marc-145 cells treated with 3000 U/mL human IFN-α (Peprotech) using QIAprep viral RNA minikit (Qiagen, Hilden, Germany) and cDNA synthesis was performed with SuperScript III Reverse Transcriptase (Invitrogen, Shanghai, China). The full-length mViperin gene was amplified with a set of primers (Table 1), and amplicons were cloned into a pEASY-Simple Blunt vector (Beijing TransGen Biotech Co. Ltd., Beijing, China) and sequenced. The determined nucleotide sequence of mViperin was compared to that found in the database (GenBank accession number: JQ437826.1). To generate the expression vector, the mViperin gene was amplified from a previously sequenced plasmid using the primers shown in Table 1. Polymerase chain reaction (PCR) products were digested with restriction enzymes and cloned into a pVAX-1 vector with the kozak sequence at the N terminus and a FLAG tag at the C terminus to produce a pVAX-mVIP plasmid. Truncations of mViperin were subcloned from the pVAX-mVIP plasmid with a FLAG tag at the N-terminus to produce pVAX-mVIP (5’Δ8, 5’Δ10, 5’Δ12, 5’Δ17, 5’Δ33 and 3’Δ143) plasmids.

**Table 1:** PCR Primers.

	Sense primer (5’-3’)	Antisense primer (5’-3’)
PCR primer		
wt-mVipern	cccaagcttgccaccatggattacaaggatgacgacgataagtgggtactcacgcctgc	Ccgctcgagctaccaatccagct tcagatcagccttact
IRF-1	atgcccatcactcggatgcgcatgaga	ctacggtgcacagggaatggcctggat
5’Δ8	cccaagcttatggactacaaggacgacgatgacaagtttgctgggaagctcctgag	ccgctcgagctaccaatccagcttcagat
5’Δ10	cccaagcttatggactacaaggacgacgatgacaaggggaagctcctgagtgtgtt	ccgctcgagctaccaatccagcttcagat
5’Δ12	cccaagcttatggactacaaggacgacgatgacaagctcctgagtgtgttcaggca	ccgctcgagctaccaatccagcttcagat
5’Δ17	cccaagcttatggactacaaggacgacgatgacaagaggcaacctctgagctctct	ccgctcgagctaccaatccagcttcagat
5’Δ33	cccaagcttatggactacaaggacgacgatgacaagtggctgagggcaacctggct	ccgctcgagctaccaatccagcttcagat
3’Δ143	cccaagcttatggactacaaggacgacgatgacaagatgtgggtactcacgcctgc	ccgctcgagctacttgaaagcgactctataat
Quantitative PCR primer		
mViperin	taaatgcggcttctgtttcc	gaaatggctctccacctgaa
PRRSVORF7	aaaccagtccagaggcaagg	tcagtcgcaagagggaaatg
IRF-1	ggctgggacatcaacaagga	gagttcatggcacagcgaaag
IFN-α	acctttgctttactggtggcc	atctgtgccaggagcatcaag
mGAPDH	gaaggtgaaggtcggagtc	gaagatggtgatgggatttc
RGAPDH	ccttcattgacctcaactacatg	cttctccatggtggtgaagac

### Plasmid transfection and virus challenge

To determine the effects of mViperin on PRRSV replication, Marc-145 cells plated on 24-well plates were respectively transfected with 0.5, 1 and 1.5 μg pVAX-mVIP or pVAX-1 plasmid DNA using Lipofectamine 3000 transfection reagent according to the manufacturer's recommendations (Invitrogen). To locate the antiviral domain of mViperin, Marc-145 cells were respectively transfected with 1 μg mViperin truncations, and pVAX-1 plasmids as described above. PRRSV strain BB0907 were infected with a MOI of 0.01 at 24 h after transfection, then the cells were analyzed by western blotting, immunofluorescence assay (IFA) and real-time PCR at an indicated time point (0, 12, 24, or 48 h) post infection (hpi). PRRSV yields in the supernatant were titrated by TCID_50_.

### Western blotting assay

Marc-145 cells treated with various methods were harvested and processed as described previously [42]. The processed protein samples were subjected to 12% sodium dodecyl sulfate–polyacrylamide gel electrophoresis (SDS-PAGE) and transferred onto nitrocellulose (NC) membrane (Pall Co., Ann Arbor, MI, USA). The membranes were blocked in Tris-buffered saline with 0.05% Tween-20 containing 10% nonfat for 2 h at 25°C and then incubated with anti-N monoclonal antibody (mAb, made in our laboratory), anti-β-actin mAb (Abmart, Shanghai, China), anti-FLAG (Abmart), overnight at 4°C. After washing three times with TBST buffer (20 mM Tris–HCl, pH 7.4, 150 mM NaCl, 0.1% Tween 20), the membranes were incubated with horseradish peroxidase- conjugated goat anti-mouse IgG (Boster Bio-Tech Co. Ltd., Wuhan, China) for 1 hour at 37°C. The signals were developed using an ECL western blotting system (Thermo Fisher Scientific, USA) and the spot levels were detected by using ImageJ quantification software.

### Quantitative reverse transcriptase PCR (qRT-PCR)

Total RNA from Marc-145 cells was extracted using total RNA kit I (Omega Bio-tek, Inc, Norcross, GA, USA) according to manufacturer’s instructions. RNA were converted to cDNA using HiScript^®^ Q RT SuperMix (+gDNA wiper) (Vazyme, Nanjing, China) according to the manufacturer’s instructions. Two microliters of the RT reaction mixture were submitted to quantitative RT-PCR (qRT-PCR) using mViperin, PRRSV ORF7, IFN-α, or IFN regulatory factor-1 (IRF-1)-specific primers (Table 1), and SYBR Green Real-time PCR Master Mix (Vazyme), according to the manufacturer’s recommendations. The reaction procedure was 95°C for 5 min, followed by 40 cycles at 95°C for 5 s and 60°C for 31 s. Standard curve analysis was performed for relative quantification. The transcript levels of target genes were relatively quantified using the 2^-ΔΔCT^ method. The *GAPDH* gene served as an internal reference. The relative amount of target gene mRNA was normalized to that of *GAPDH* mRNA in the same sample.

### Indirect immunofluorescence assay (IFA)

Marc-145 cells plated on 12-well plates with different treatments were washed three times with phosphate-buffered saline (PBS) and fixed with methanol at 4°C for 45 min. PRRSV N protein was detected as described previously using a monoclonal antibody to N protein of PRRSV (made in our laboratory) [42]. The nuclei were stained using 4’,6-diamidino-2-phenylindole (DAPI; Invitrogen, China).

### mViperin siRNA knockdown

The mViperin siRNA (S1 and S2) and negative control siRNA (siNC) (Invitrogen) were transfected with Lipofectamine 3000 transfection reagent (Invitrogen). Marc-145 cells with 80% confluence on a 24-well plate were transfected with 60 pmol mViperin siRNA and siNC for 6 h prior to administration of 1000 U/mL IFN-α (Peprotech). Twenty-four hours later, cells were infected with PRRSV at a MOI of 0.01 for 48 h. The effect of siRNAs and IFN-α on mViperin expression and PRRSV replication were detected by using qRT-PCR, western blotting and virus yield titration. The siRNA sequences were: S1: 5′-gcaacuauaaaugcggcuutt-3′; S2:5′-gggugagaauuguggagaatt-3′. siNC, 5′-uucuccgaacgugucacgu-3′ (scrambled oligonucleotides).

### Internalization assay

The effect of mViperin suppression on PRRSV internalization was also evaluated by western blot assay. Marc-145 cells with 80% confluence were washed and cultured with serum-free DMEM medium for 12 h. Then the cells were incubated with PRRSV at 1 MOI for 1 h at 4°C to allow virus attachment without internalization. The cells were washed with ice-cold PBS three times so that unbound viruses were removed. Then the culture medium was replaced with fresh serum-free DMEM and the cells were subsequently shifted to 37°C with 5% CO_2_ to allow virus internalization. The cells were washed with citrate buffer solution (pH = 3) to remove the non-internalized visions on the surface of cells, and then the cells were washed with ice-cold PBS three times. The level of viral protein in cells was detected by using a western blot assay [43].

### Co-immunoprecipitation assay

Marc-145 cells were transfected with pVAX-mVIP or pVAX-1 using lipofectamine 3000 (Invitrogen). At 24 h post transfection (hpt), the cells were infected with PRRSV BB0907 (MOI = 1). Then 36 h later, the cells were rinsed three times in cold PBS. Cells were then lysed in protein extraction reagent for 30 min on ice, followed by centrifugation at 5000×g for 10 min at 4°C to remove cell debris. Cell extracts were incubated with rabbit anti-FLAG polyclonal antibody (Cell Signaling, Boston, MA, USA) or mouse anti-PRRSV N (made in our laboratory), GP5 mAb (made in our laboratory) for 12 h at 4°C, then A/G-agarose beads (Beyotime, Shanghai, China) were added. After 4 h incubation, the beads were collected by centrifugation at 2500 g for 5 min and washed five times with cold PBS. The beads were boiled in 2×SDS loading buffer to elute bound protein and subjected to western blotting; the proteins were analyzed by mouse anti-FLAG mAb (Abmart, Shanghai, China) or rabbit polyclonal antibody (Cell Signaling), and mouse anti-PRRSV N protein mAb (made in our laboratory).

### Confocal microscopy analysis

Marc-145 cells plated onto a cover glass in a 24-well plate were transfected with pVAX-mVIP or pVAX-1. Twenty hours later, the cells were infected with PRRSV BB0907 (MOI = 1) and incubated for 36 h. After being fixed with 1:1 methanol/acetone, the cells were incubated with mouse anti-PRRSV N, GP5 (made in our laboratory) and rabbit anti-FLAG-mViperin antibodies (Cell Signaling), mouse anti-calnexin (Cell Signaling) for 1 h at 37°C. After being washed three times, the cells were incubated with a mixture of Alexa Fluor 555-conjugated donkey anti-rabbit (Beyotime) and FITC-conjugated goat anti-mouse secondary antibodies (Boster) for 1 h at 37°C. The nuclei were stained using DAPI, and cover slips were mounted onto a slide glass using 10% glycerol. After three washes, confocal images were obtained using a Zeiss LSM 710 scanning confocal microscope.

### Statistical analysis

The results were analyzed for significance by one-way or two-way analysis of variance using GraphPad Prism for Windows version 5.02 (GraphPad, San Diego, CA, USA). P<0.05 indicated significant differences between two groups.

## Results

### mViperin mRNA is upregulated by IFN-α and PRRSV infection

**Fig 1:**
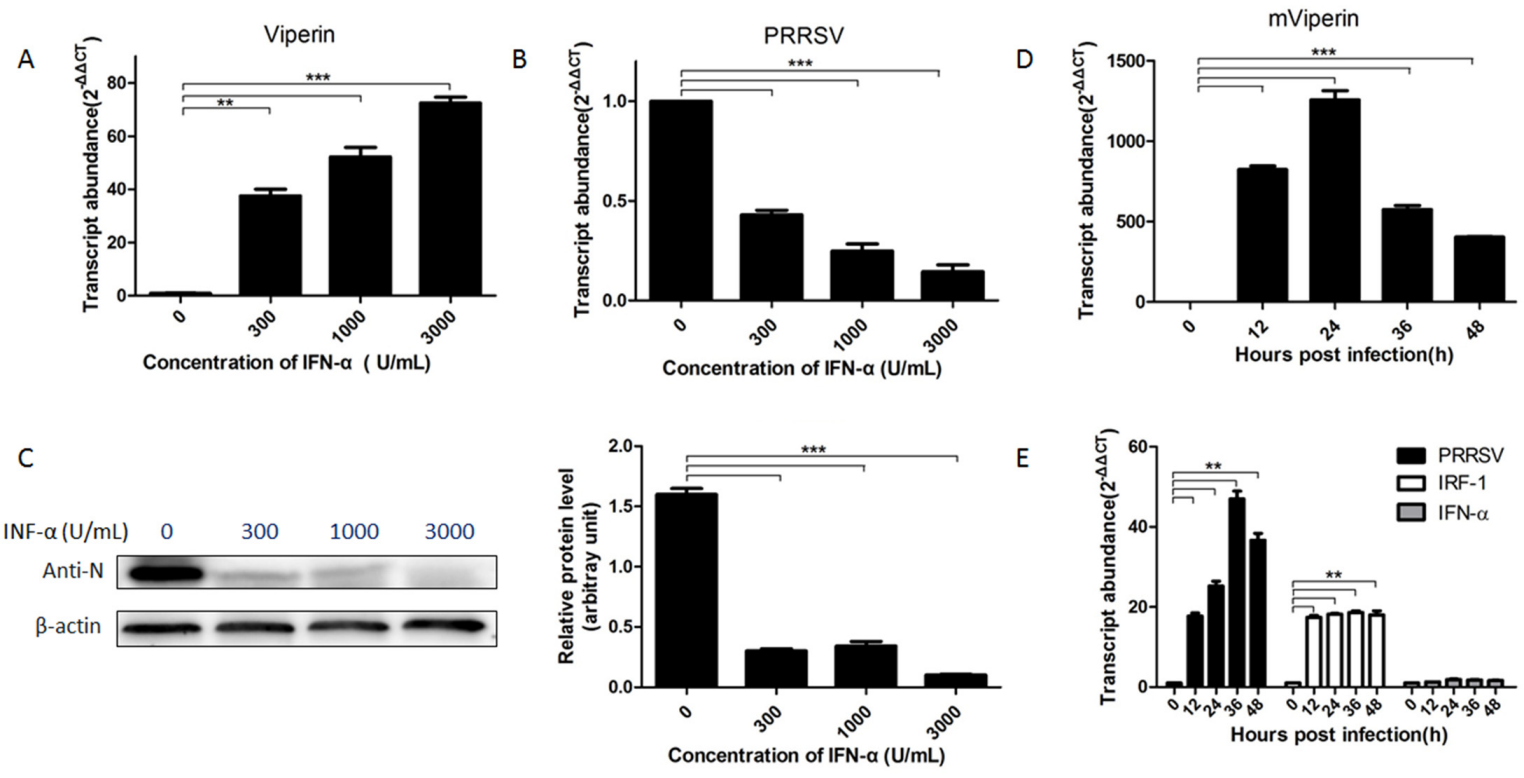
Induction of mViperin and inhibition of PRRSV by IFN-α is dose-, and time-dependent. Marc-145 cells were stimulated with IFN-α at concentrations of 0–3000 U/mL for 24 h, and infected with PPRSV BB0907 at 0.1 multiplicity of infection (MOI). At 48 h post infection, PRRSV-ORF7 and mViperin mRNA were detected with real-time PCR, and the levels were normalized to the level of mRNA (A, B). Meanwhile, PRRSV was examined by western blotting with anti-PRRSV N protein. The levels of PRRSV N protein were then quantified by immunoblot scanning and normalized with respect to the amount of β-actin (C). Marc-145 cells were infected with PRRSV BB0907 (at 1 MOI) then, at the indicated time points, the cells were harvested and mViperin mRNA, PPRSV and IRF-1, IFN-α mRNA were detected by quantitative RT-PCR, the levels were normalized to the level of GAPDH mRNA (D, E). The values represent mean ± SEM. *, **, *** indicates a significant difference of P < 0.05; P < 0.01; P < 0.001, respectively.

To investigate the expression of mViperin in Marc-145 cells during PRRSV infection, Marc-145 cells were infected with the PRRSV BB0907 strain and mViperin mRNA expression was detected by qRT-PCR and western blotting assay. The results showed that mViperin expression levels were significantly increased by treatment with IFN–α in a dose-dependent manner (Fig 1A). Meanwhile, PRRSV infection was obviously decreased after IFN-α treatment at 300–3000 U/mL concentration (Fig 1B and 1C). The peak expression levels of mViperin mRNA arrived at 24 hpi, as shown in Fig 1D.

To understand the effect of IRF-1 on induction of mViperin by PRRSV BB0907, IRF-1 mRNA levels were detected with qRT-PCR. The results showed that expression levels of mViperin and IRF-1 in Marc-145 cells infected with PRRSV BB0907 strain are simultaneously increased compared with those without the virus infection. Meanwhile, the IFN-α mRNA levels were detected with qRT-PCR. The results showed that no significant changes occurred for IFN-α mRNA at different time points in Marc-145 cells infected with PRRSV, indicating that the expression of mViperin is independent of IFN-α production (P<0.05, Fig 1E).

### mViperin inhibits PRRSV replication

**Fig 2:**
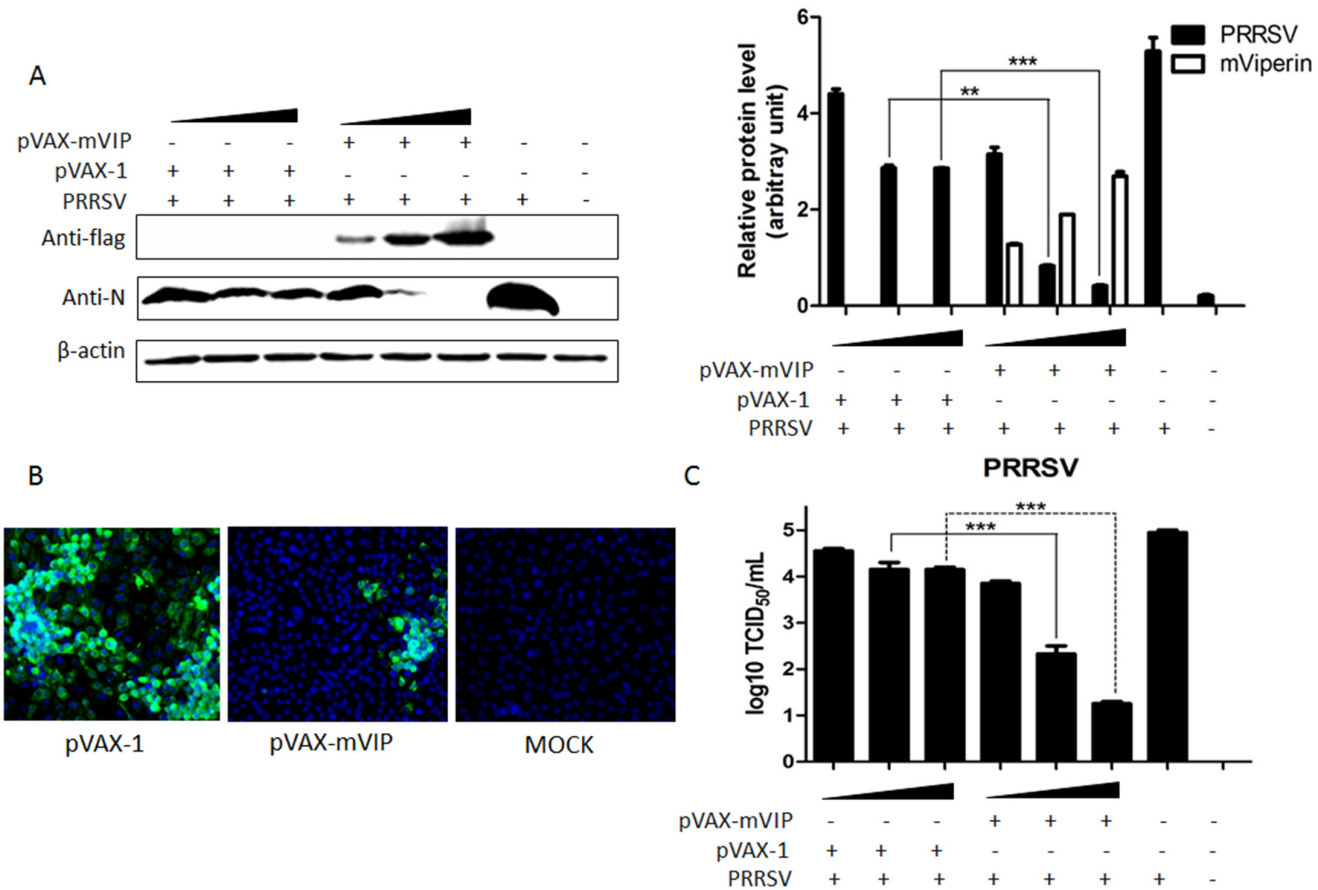
Overexpression of mViperin inhibits PRRSV replication in a dose-dependent manner. Marc-145 cells were transfected with 0.5, 1, 1.5 μg of pVAX-mVIP or pVAX-1 plasmids, individually. After being transfected for 24 h, the cells were subsequently infected with 0.01 multiplicity of infection (MOI) PRRSV BB0907. At 48 h post infection, the cells were harvested and the PRRSV and mViperin was detected by western blotting with anti-N and anti-FLAG antibodies. The levels of mViperin and N protein were quantified by immunoblot scanning and normalized with respect to the amount of β-actin (lower panel) (A). Meanwhile, PRRSV in the cells was detected by IFA (B), and the virus yields in the culture supernatants were detected by TCID_50_ (C). Values represent mean ± SEM. *, **, *** indicate a significant difference of P < 0.05; P < 0.01; P < 0.001, respectively. Data represent one of two independent experiments.

To determine if mViperin plays a role in inhibiting replication of PRRSV, Marc-145 cells were transfected with pVAX-mVIP and pVAX-1 plasmids at 0.5 μg, 1 μg and 1.5 μg doses and then infected with PRRSV BB0907 (MOI = 0.01). At 48 hpi, the cells were harvested and levels of PRRSV N protein in the cell lysates were examined by western blotting assay, and PRRSV yield titer in the culture supernatant were detected. The results demonstrated that overexpression of mViperin inhibited PRRSV replication in a dose-dependent manner (Fig 2A and 2C). Meanwhile, IFA results showed that the number of infected cells in the pVAX-mVIP transfection group were obviously less than that in the pVAX-1 transfection group (Fig 2B).

Meanwhile, to further investigate whether the inhibition effect of mViperin is related to the amount of inoculated PRRSV, the Marc-145 cells transfected with 1 μg pVAX-mVIP were infected with various amounts of PRRSV (MOI = 0.01,0.1,1). The result indicated that the anti-PRRSV effect of mViperin is significant when the cells were inoculated with PRRSV at a MOI of 0.1, 0.01. Moreover, the anti-PRRSV effect of mViperin was lost with an increase of infected PRRSV MOI to 1. The results showed that anti-viral functions of mViperin depend on virus titers.

### Knockdown of mViperin enhances PRRSV replication and impairs the antiviral activity mediated by IFN

**Fig 3:**
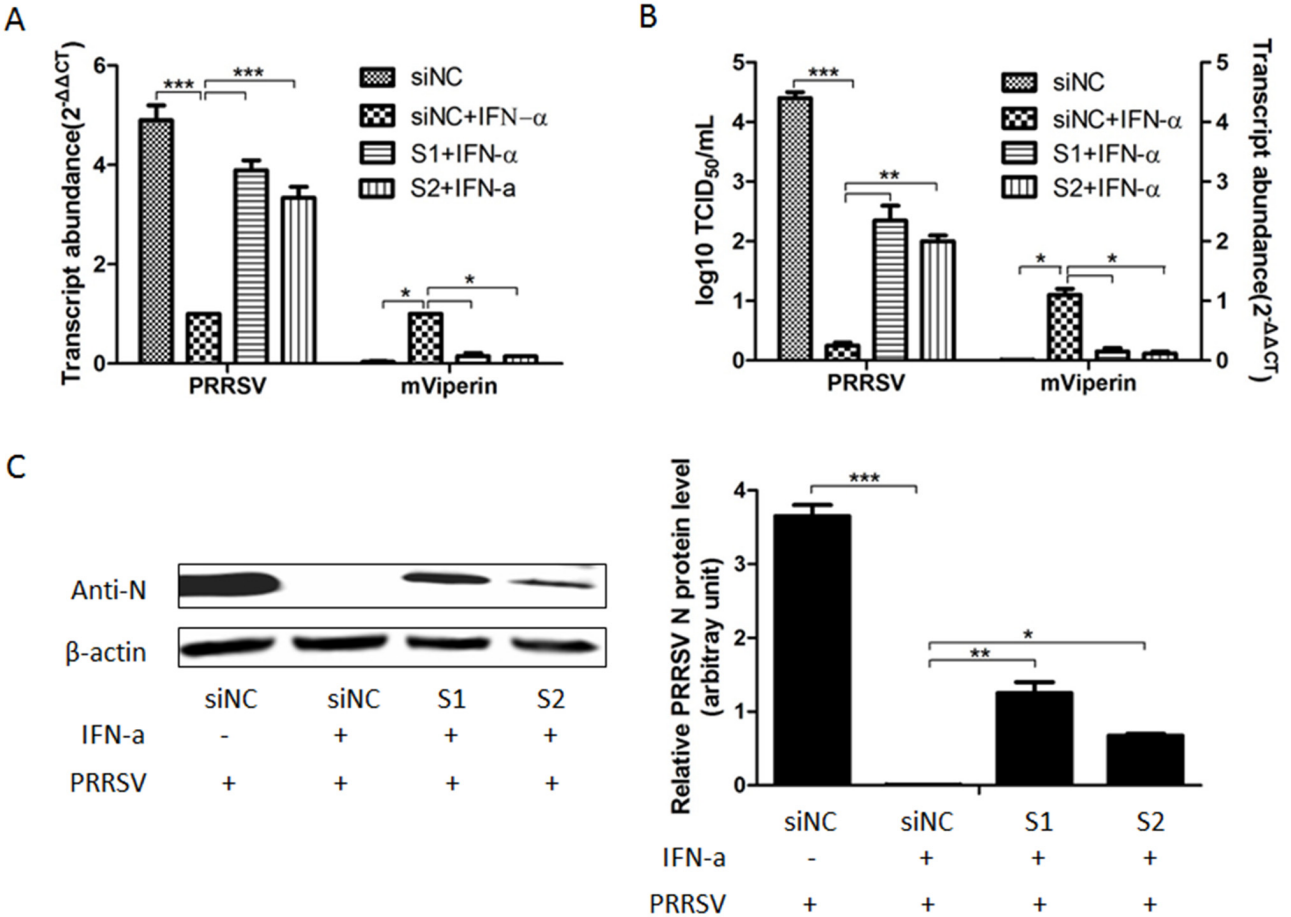
Knockdown of mViperin increased PRRSV replication and impaired IFN-α-mediated antiviral activity. Marc-145 cells in six wells of the 24-wells-plate were transfected with S1, S2 and siNC, and 2% DMEM containing 800 U/mL of IFN-α-2a was added, after 24 h two wells of each group were harvested and mViperin mRNA levels were detected by QPCR (A, B). Another four wells were infected with 0.01 multiplicity of infection (MOI) PRRSV BB0907. At 48 h post infection, the total RNA in the 2 well cells in each group was extracted for the determination of PRRSV ORF7 mRNA levels by quantitative RT-PCR and the levels were normalized to the level of *GAPDH* mRNA in the same sample (A). Meanwhile, the culture supernatants were collected and the virus yields were detected by TCID_50_ (B). The two well cells in each group were harvested for western blotting analysis with anti-N and anti-β-actin antibodies. The levels of N protein were quantified by immunoblot scanning and normalized with respect to the amount of β-actin (lower panel) (C). Values represent mean ± SEM; *, **, *** indicate a significant difference at P < 0.05; P < 0.01; P < 0.001, respectively.

To determine if mViperin expression is necessary to control PRRSV replication, Marc-145 cells were transfected with mViperin-specific siRNA S1 and S2, and negative siRNA control siNC before administration with 800 U/mL of IFN-α, and then were infected with the PRRSV BB0907 strain. QRT-PCR assay results showed that the expression of mViperin mRNA was significantly decreased by mViperin siRNA compared to those by siNC in Marc-145 cells after being administrated with IFN-α (Fig 3A and 3B). The PRRSV ORF7 mRNA levels and viruses yields in S1 and S2 treatment groups were significantly higher than those in the siNC group (Fig 3A and 3B). Meanwhile, western-blot results also showed that knockdown of mViperin mRNA by siRNA significantly enhanced the production of PRRSV in Marc-145 cells (P<0.05) (Fig 3C). This indicates that mViperin is necessary for IFN-α-mediated anti-PRRSV effects in Marc-145 cells.

### mViperin blocks an early step of PRRSV entry and genome replication but does not affect virus assembly or release

**Fig 4:**
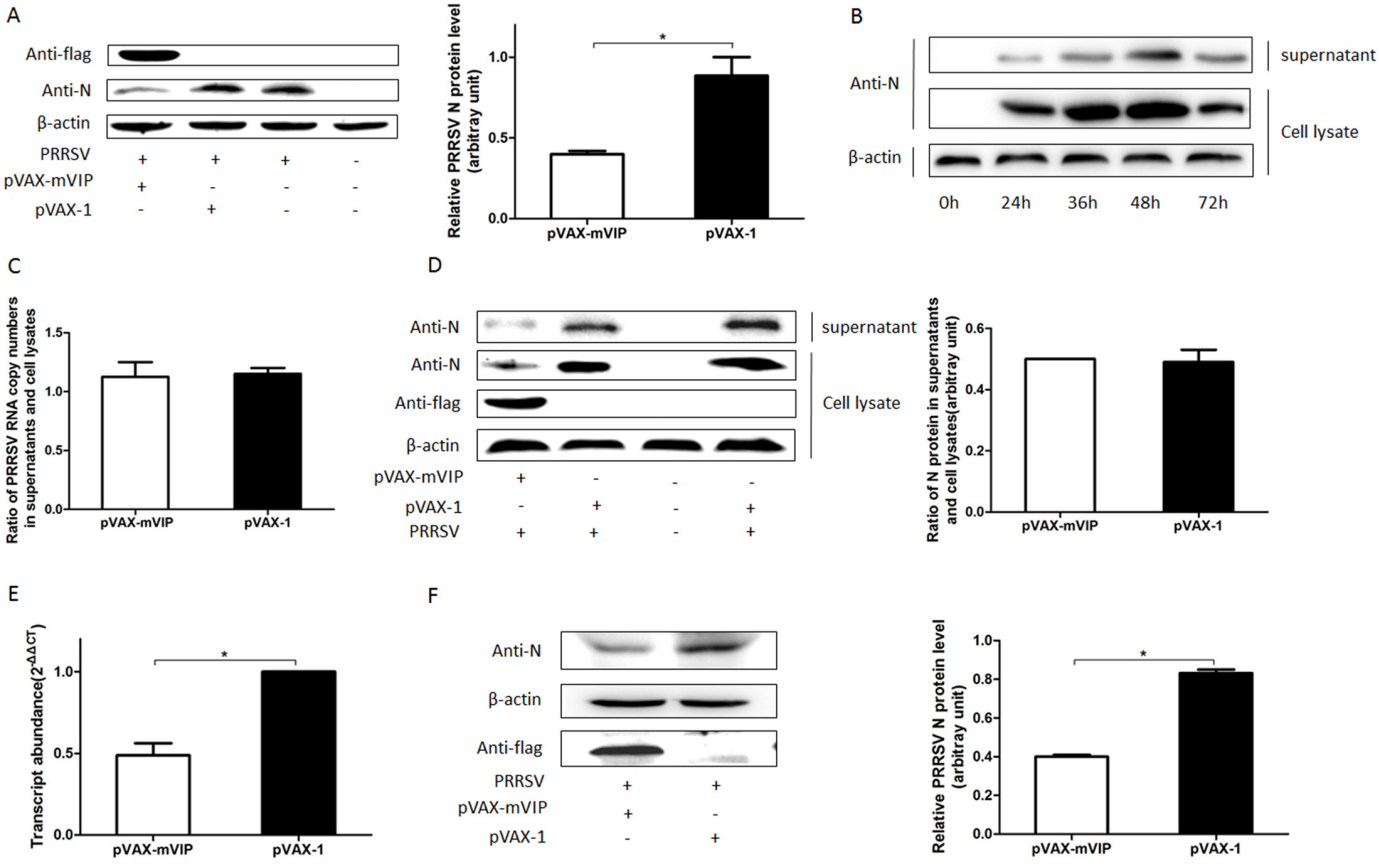
mViperin blocks an early step of PRRSV entry but does not affect virus assembly or release. Marc-145 cells were transfected with pVAX-mViperin and pVAX-1, after being transfected for 24 h, the cells were infected with one multiplicity of infection (MOI) PPRSV BB0907 at 4°C for 1 h, after washing with cold PBS, the infected cells were further incubated at 37°C for 1 h, the bound but non-internalized virus particles were removed and washed with citrate buffer solution (pH = 3). After washing with cold PBS, the cells were harvested for western blotting with anti-N, anti-FLAG and anti-β-actin antibodies. The levels of mViperin and N protein were quantified by immunoblot scanning and normalized with respect to the amount of β-actin (lower panel) (A). Marc-145 cells were infected with 0.01 MOI PRRSV BB0907. At indicated times the ratio of the virus N protein in supernatants and cell lysates were determined by western blotting (B). In another experiment, after being transfected with pVAX-mViperin and pVAX-1, Marc-145 cells were infected with PRRSV (MOI = 0.01). At 48 h post infected with PRRSV, the ratio of PRRSV RNA copy number between the supernatants and the cell lysates was detected by Q-PCR (C), and the ratio of the virus N protein in supernatants and cell lysates was determined by western blotting (D). BHK21 cells were transfected with 1 μg of pVAX-mVIP or pVAX-1 plasmids, individually. After being transfected for 24 h, the cells were subsequently transfected with 1 ug of PRRSV strain BB0907 infectious cDNA clone. At 48 h post transfection, the cells were harvested and PRRSV ORF7 mRNA and N protein and mViperin was detected by quantitative RT-PCR (E) and western blotting (F). Values represent mean ± SEM; *, **, *** indicated a significant difference of P < 0.05; P < 0.01; P < 0.001, respectively.

To analyze the stages of the dissected virus life cycle affected by mViperin, the earliest virus entries were initially assessed by internalization assay. Marc-145 cells seeded on 24-well plates were transfected with pVAX-mVIP and pVAX-1, and then incubated with the PRRSV BB0907 strain at 4°C for 1 h to permit virus attachment and further held at 37°C to allow virus internalization, followed by washing with PBS and citrate buffer solution to remove the remaining viruses. And the cells were harvested to detect the PRRSV N protein in cell lysate by western blot. The results showed that the amount of PRRSV that entered the cells transfected with pVAX-mVIP was decreased compared to those transfected with pVAX-1, suggesting that mViperin inhibited PRRSV entry to the host cell (Fig 4A).

To further detect the antiviral effect of mViperin on the stages of PRRSV assembly or release, Marc-145 cells were transfected with pVAX-mViperin or pVAX-1, and then incubated with PRRSV for 48 h and the cells and supernatants were collected for western blot and real-time PCR assay [44]. The results showed the ratios of the RNA level of PRRSV in supernatant to cell lysates were almost the same between pVAX-mViperin and pVAX-1 transfected cells, indicating that overexpression of mViperin did not affect virus assembly. Meanwhile, although the result showed that the amount of N protein in cell lysates were almost 3-fold higher than that in the cultural supernatants, the ratio between them was not obviously different between the pVAX-mViperin or pVAX-1 groups. These results suggest that mViperin had no effect on viral release (Fig 4B, 4C and 4D).

To investigate whether Viperin inhibits PRRSV infection at the levels of viral genomic replication and translation, BHK21 cells, which have no PRRSV receptors but allow the replication and translation of the PRRSV genome [45, 46], were co-transfected with pVAX-mVIP and the plasmid DNA containing PRRSV strain BB0907 infectious cDNA. At 48 hpt, the cells were harvested and PRRSV ORF7 mRNA and N protein were detected by qRT-PCR and western blot. The results showed the intracellular levels of PRRSV RNA and N protein were significantly lower than those in the pVAX-1 control group (Fig 4E and 4F). These results indicate that mViperin could inhibit PRRSV genome replication and translation.

### mViperin co-localizes with both PRRSV N and GP5 proteins and interacts with N protein in Marc-145 cells

**Fig 5:**
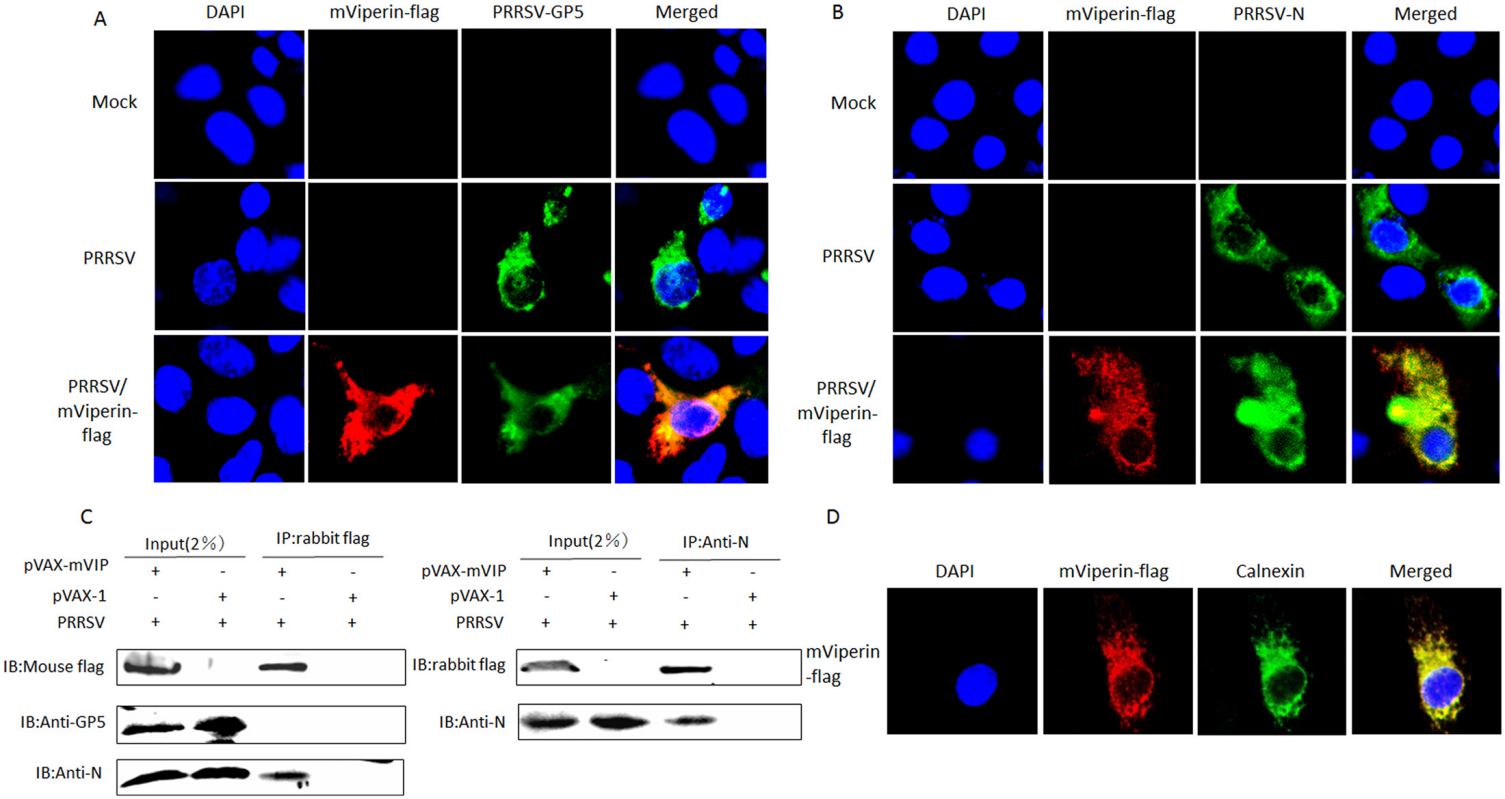
Co-localization of mViperin and PRRSV N or GP5 proteins in Marc-145 cells. Marc-145 cells were transfected with pVAX-mVIP, after being incubated for 24 h, the cells were infected with PRRSV BB0907 (MOI = 1). At 36 h post infection (hpi), the cells were fixed with 1:1 methanol/acetone and the mViperin and PRRSV N and GP5 were detected using anti-FLAG and anti-PRRSV N (A) or GP5 (B) protein antibodies, followed by incubation with a mixture of Alexa Fluor (red)-conjugated donkey anti-rabbit and 488 (green)-conjugated goat anti-mouse secondary antibodies. The nuclei were stained with DAPI stain (blue), and expression was analyzed by confocal microscopy. (C) mViperin and N interaction by immunoprecipitation. Marc-145 cells plated onto six-well plates were transfected with or without 4 μg pVAX1-VIP and incubated for 24 h. Cells were infected with or without PRRSV BB0907 strain (1 MOI). At 48 hpi, the cell lysates were immunoprecipitated with anti-FLAG or anti-PRRSV N or GP5 protein antibodies and subjected to western blotting with antibodies to FLAG and PRRSV N protein. Meanwhile, the co-localization between mViperin and ER marker calnexin were detected by anti-FLAG-mViperin and anti-calnexin antibodies (D).

PRRSV GP5 protein is an important virus structural protein, and plays a key role in cell recognition and binding, apoptosis, and immune protection [47, 48]. The GP5 protein localizes to the ER in the process of PRRSV assembly and maturation, and a GP5 and M heterodimer is formed on the ER for transport to the Golgi complex [48, 49]. Meanwhile N protein is also important in the assembly and replication of PRRSV and often considered an indicator of viral replication. To determine if mViperin could interact with PRRSV N and GP5 in the process of PRRSV replication, the distribution of mViperin, PRRSV GP5 and N protein were initially assessed in Marc-145 cells infected with PRRSV. Marc-145 cells transfected with pVAX-mViperin or pVAX-1 were infected with PRRSV BB0907, and GP5, N protein and mViperin were detected by confocal microscopy assay using mouse anti-PRRSV N, GP5 and rabbit anti-flag antibodies. The results revealed that mViperin considerably co-localized with GP5 and N protein in Marc-145 cells. Meanwhile, overexpressed mViperin also colocalized with the endoplasmic reticulum (ER) marker calnexin intracellularly, and this is consistent with the distribution of Viperin protein of other species (Fig 5A, 5B and 5D).

To further verify the interactions between mViperin and PRRSV GP5 and N protein, co-immunoprecipitation (Co-IP) experiments were performed by transfection of FLAG-tagged mViperin expression plasmids in Marc-145 cells and infection with PRRSV BB0907. The result showed that mViperin interacted with PRRSV N protein, but did not interact with GP5 protein (Fig 5C).

### The N-Terminal of mViperin is necessary and sufficient for antiviral activity of mViperin

**Fig 6:**
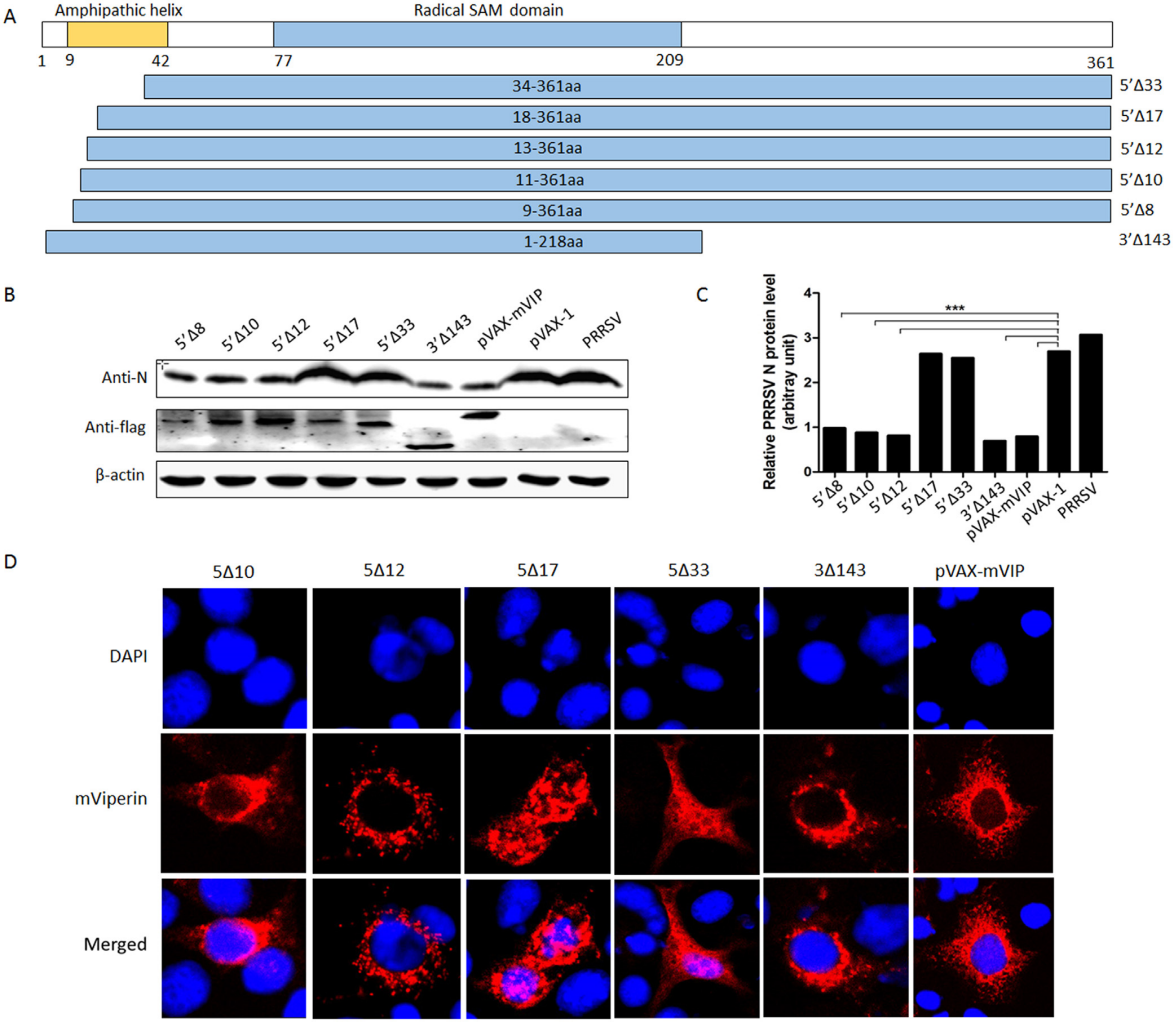
The N-terminal regions of the mViperin protein are required for its anti-viral activity. **(A**) Schematic diagram of mViperin and mutant derivatives. (B) Marc-145 cells were transfected with the plasmids expressing mutants or wild type mVipeirn. At 24 h post transfection, the cells were infected with PRRSV BB0907, and at 48 h post infection (hpi), the cells were harvested for western blotting with anti-N, anti-FLAG and anti-β-actin antibodies. The levels of mViperin and N protein were quantified by immunoblot scanning and normalized with respect to the amount of β-actin (lower panel). (C) Marc-145 cells were transfected with plasmid expressing WT mViperin or mViperin mutants, and infected with PRRSV. At 36 hpi, the cells were fixed with 1:1 methanol/acetone and detected using anti-FLAG antibodies, followed by incubation with a mixture of Alexa Fluor (red)-conjugated donkey anti-rabbit and 488 (green)-conjugated goat anti-mouse secondary antibodies. The nuclei were stained with DAPI stain (blue), and expression was analyzed by confocal microscopy.

To identify the functional domain of mViperin required for inhibiting PRRSV replication, a series of mViperin amino-terminal truncations were constructed in this study as shown in Fig 4. Marc-145 cells were respectively transfected with truncated mViperin plasmid DNA as described above. As shown in Fig 6, the N-terminal truncation in mViperin (5’Δ12, 5’Δ10, 5’Δ8) retained the same antiviral activity as the parent mViperin. In contrast, truncated mViperin (5’Δ17, 5’Δ33) were inactive. Meanwhile, all truncated mViperin proteins were confirmed to be well expressed by immunoblotting using anti-FLAG-tag. This indicated that the 13–16 amino acids of mViperin plays an important role in suppressing PRRSV replication. In addition, the C-terminal truncation mViperin (3’Δ143) still inhibits PRRSV replication, suggesting that the C-terminal region is not necessary for mViperin antiviral activity.

Meanwhile, we observed the effects of the N- and C-terminal of mViperin on the distribution of mViperin in Marc-145 cells by confocal microscopy assay. The results showed that the truncation of mViperin (5’Δ17, 5’Δ33) changed the location of mViperin in the cytoplasm to the entire intracellular region as a granular to uniform dispersal (Fig 6C). This indicated that the 17 amino acids from the mViperin N-termini played an important role for the distribution of the protein in the cell.

## Discussion

Viperin can be induced by type I, II and III IFN, double-stranded DNA, or double-stranded RNA analogues, and inhibits the replication of many viruses by apparently diverse mechanisms [21, 28, 29, 32, 50, 51]. PRRSV is known to inhibit the synthesis of type I IFNs in infected pigs, and results in the suppression of innate immunity [51–53]. Current vaccination strategies cannot control this infectious disease. Antiviral therapy should be an effective supplement for the control of PRRSV infection. However, the role of Viperin in PRRSV infection is scarcely understood. Furthermore, viperin can be up-regulated by both IFN-dependent and IFN-independent pathways [17, 18, 23, 28, 29, 34, 36, 50, 54]. Stirnweiss et al. described that IFN-independent induction of viperin is mediated via IRF-1 during infection of vesiculars tomatis virus (VSV) [17, 18, 23, 28, 29, 34, 36, 50, 54]. The virus infection can strongly up-regulate viperin gene transcription by the STAT-IRF1-Viperin pathway [18]. Another report showed that human cytomegalovirus infection can activate the type I IFN signaling pathway, which enhances viperin transcription through the STAT1/STAT2/IRF-9 complex termed ISG factor 3 (ISGF3) by binding to the promoter response element ISRE [55, 56]. JEV was able to induce viperin expression through IRF-3 and AP-1. In turn, JEV infection utilized the proteasome-mediated pathway to degrade the viperin protein for antagonizing the host innate immunity and this function depends on N-linked glycosylation [32]. In this study, our results show that mViperin was up-regulated in response to infection with PRRSV in Marc-145 cells, but the level of IFN-α expression did not change in the cells after infecting with PRRSV, suggesting mViperin is induced via an IFN-independent pathway. The induction of mViperin may depend on IRF-1. The exact signaling pathway should be studied in the future.

Meanwhile, our results demonstrate that the expression of mViperin protein was induced by type I-IFN-α in a dose- and time-dependent manner, and it could inhibit PRRSV replication in Marc-145 cells. Overexpression of mViperin inhibited PRRSV replication in a dose-dependent manner, whereas knockdown of endogenous mViperin largely recovers PRRSV replication inhibited by IFN-α. These results suggest that mViperin is an ISG that plays an important role in the anti-PRRSV activity of IFN-α.

Viperin is a radical S-adenosylmethionine (SAM) domain-containing 2 (RSAD2) enzyme that is comprised of 361 amino acids and has a molecular mass of approximately 42 kDa [57, 58]. It is anchored to the ER and lipid bodies via a α-helix to induce ER membrane curvature. The C-terminal of viperin is conserved across species and is relevant in inhibiting the replication of DENV and HCV [22, 29, 59, 60]. The center region of viperin is highly homologous with the MoA motif of SAM enzymes, and is essential in affecting Bunyamwera virus replication and suppressing the HIV egress from cells [26, 34]. The N-terminal of viperin contains an amphipathic α-helix that is variable among different species, but leucine residues are not conserved. Some previous studies have shown that the α-helix domain is not related to antiviral function against HCV, DENV, WNV and HIV [23, 29, 33]. However, another report pointed out that the N-terminal of viperin was sufficient to suppress the infection of CHIKV, and this function is related to the localization of viperin to the ER and lipid droplets [38]. In this study, we also demonstrated that the C-termini of mViperin has no effect on PRRSV replication by detecting the anti-PRRSV activity of mutant 3’Δ143. And deletion of the 17, 33 and 50 amino acids from the N-termini significantly abrogated its anti-PRRSV activity individually, suggesting the 13–16 amino acids of the N-termini play an important role in anti-PRRSV replication.

Viperin inhibits virus replication by different mechanisms. For example, viperin inhibited the synthesis of HCMV-encoded viral protein pp65, gB and pp28 and is redistributed to exert its antiviral effect [17]. Moreover, viperin also disturbed the interaction between host protein hVAP-33 and HCV NS5A through binding to hVAP-33 to impact HCV replication at the RNA level [23]. Furthermore, eViperin distorted ER and damaged protein transportation in intracellular regions, reducing the efficiency of exporting from cell membrane to suppress the egress of EIAV Gag protein and inhibit the expression of EIAV Env and its receptor [28]. Here, our results showed that mViperin co-localizes with endoplasmic reticulum (ER) marker calnexin. And the mViperin could interact with the PRRSV N protein. In addition, co-transfection of pVAX-mViperin and PRRSV infectious clones in BHK21 cells showed that mViperin also could inhibit the replication and translation of the PRRSV genome. This suggests the interaction between mViperin and PRRSV N affected the synthesis and functions of the PRRSV genome and structural proteins to suppress PRRSV replication. Of course, further studies of the viral-host interactions are needed. Meanwhile, we also noted that deletion of the 33 amino acids from the N-termini shifted the Viperin distribution from a granular distribution in the cytoplasmic into a uniform dispersal into the entire intracellular region [61]. The deletion of the 17 amino acids from the N-termini changed the distribution but not the granular morphology. The complete α-helix might be necessary for localization and antiviral effects.

Previous studies have demonstrated that viperin suppressed the expression of membrane-targeting proteins, the EIAV Env protein and virus receptor protein ELR1, blocks the entry of EIAV [28]. Viperin can bind and inhibit farnesyl diphosphate synthase (FPPS), which is essential for isoprenoid biosynthesis, and results in an effect on the formation of lipid rafts which play an important role in inhibiting the egress and release of HIV and influenza [25, 26]. It has been proven that lipid rafts, existing in cell plasma membranes, are associated with the receptor CD163 that interacts with PRRSV GP3 and GP4 to mediate the internalization and fusion of PRRSV [62]. Meanwhile, cholesterol is also a component of PRRSV virions to sustain integrity, and depletion of cholesterol, a key component of lipid raft microdomains, could inhibit PRRSV infectivity due to virion disruption, loss of capsid protein from virions, and blocking of membrane fusion. In the meantime, there was no obvious difference in the release of PRRSV when lipid rafts were disrupted, but the abnormal PRRSV particles were enhanced [63, 64]. It has been reported that reducing FPPS level affects the membrane fluidity to control the HIV and influenza virus infection [25, 26]. HIV infection led to viperin protein redistribution from ER to CD81 compartments [26]. Here, we found that overexpression of mViperin blocked an early step of PRRSV entry, but did not inhibit assembly and release. In view of the above-mentioned functions of lipid raft following PRRSV infection, it is possible that mViperin suppresses the formation of cells and virus lipid rafts to alter virion components and damage integrity, inhibiting the membrane fusion to impact the infectivity and entry of PRRSV. The interaction between mViperin and CD163 or GP2/GP3/GP4, and the exact mechanism of mViperin targeting to N protein to inhibit PRRSV replication or internalization should be studied in the future.

To summarize, in this study it was first found that IFN-α induced mViperin could inhibit PRRSV replication by blocking the early steps of PRRSV entry and genome replication and translation. It could interact with N proteins in distinct cytoplasmic loci. The major antiviral activity determinant residues were located within the 13–16 amino acids of mViperin, and the N-termini of mViperin determines the distribution of mViperin protein in the cells. These findings should be useful for the future development of novel antiviral therapies against PRRSV infection.

## Supporting Information

S1 Fig Detection of the mVipeirn expression in Marc-145 cells transfected with pVAX-mVIP. Marc-145 cells were transfected with 1 μg of pVAX-mVIP, and mVipeirn in the cells was detected by IFA with anti-flag antibodies.
(TIF)
