# Supplementary figures and images for: Monkey Viperin Restricts Porcine Reproductive and Respiratory Syndrome Virus Replication
Source: PLoS One. 2016 May 27;11(5):e0156513. doi: 10.1371/journal.pone.0156513 (PMC4883763; doi:10.1371/journal.pone.0156513)

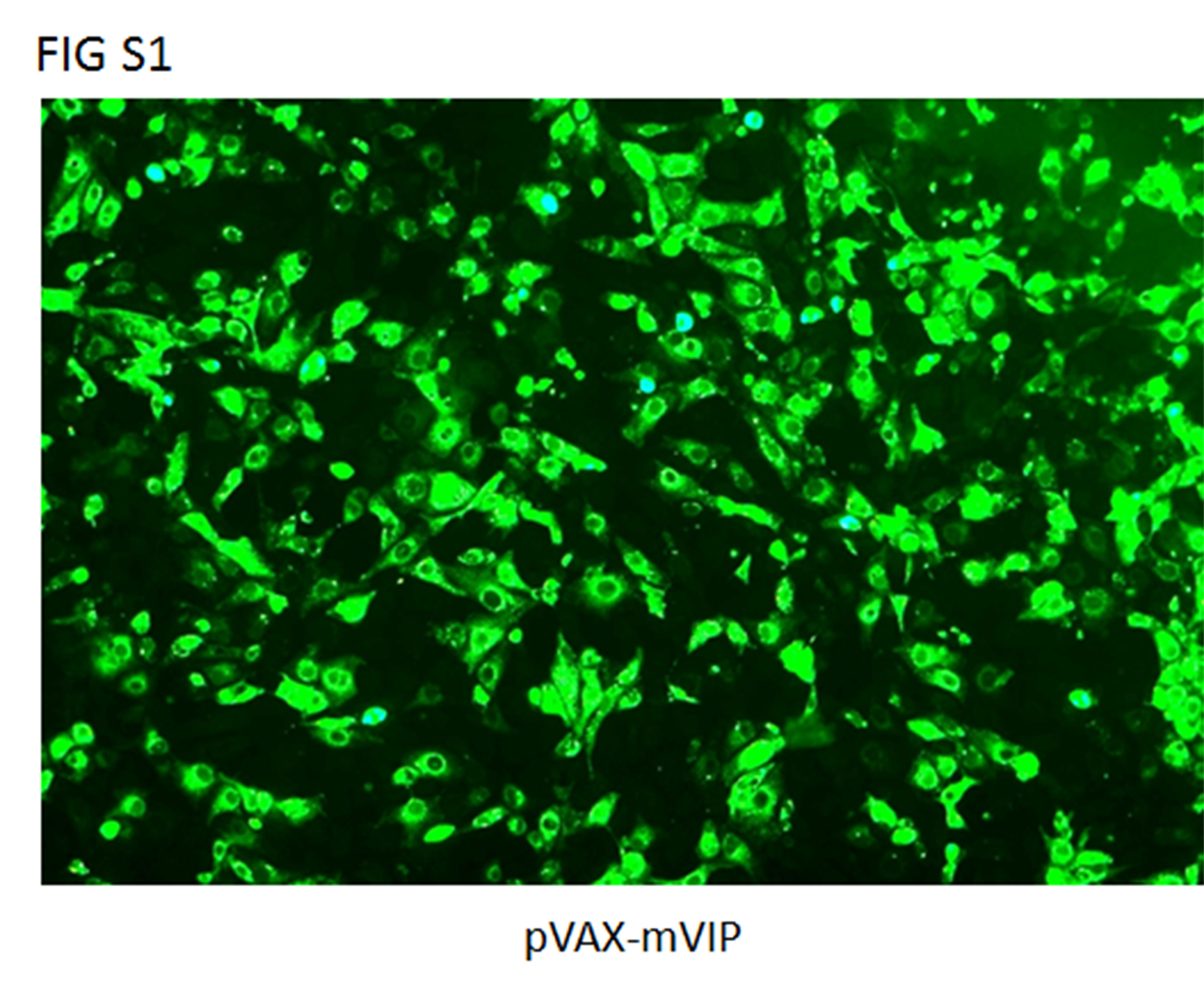

Supplement: S1 Fig — Marc-145 cells were transfected with 1 μg of pVAX-mVIP, and mVipeirn in the cells was detected by IFA with anti-flag antibodies. (TIF) [file pone.0156513.s001.tif]
